# Medically-attended anxiety and depression is increased among newly diagnosed patients with cold agglutinin disease: Analysis of an integrated claim-clinical cohort in the United States

**DOI:** 10.1371/journal.pone.0276617

**Published:** 2022-12-15

**Authors:** Catherine M. Broome, Naushin Hooda, Jun Su, Xiaohui Jiang, Gina Nicholson, Cara L. Frankenfeld, Melitza Iglesias-Rodriguez, Jon Fryzek, Parija Patel

**Affiliations:** 1 Division of Hematology, MedStar Georgetown University Hospital, Washington, DC, United States of America; 2 EpidStrategies, Rockville, Maryland, United States of America; 3 Sanofi, Cambridge, Massachussetts, United States of America; Istanbul University-Cerrahpaşa, Cerrahpaşa Faculty of Medicine, TURKEY

## Abstract

**Background:**

Cold agglutinin disease (CAD) is a rare, chronic form of autoimmune hemolytic anemia. Clinical manifestations can include classical complement pathway-mediated chronic hemolysis, anemia, and profound fatigue. Research has shown that patients with other anemias may develop anxiety and depression, but this has not been studied previously in patients with CAD.

**Methods:**

CAD patients were identified in the Optum Claims-Clinical dataset (between January 1, 2006–June 30, 2016) and matched to comparison patients without CAD by patient factors. Adjusted Cox regression models estimated time to anxiety and depression, defined by three different outcomes: medication use, hospitalization, and therapy related to anxiety and depression. Subset analyses were performed for primary CAD. Patients were followed until they had anxiety and depression, they left the Optum system, death, or the study period ended (June 30, 2016).

**Results:**

Patients with CAD (n = 384) were more likely to have medically attended anxiety and depression (adjusted hazard ratio [aHR]: 1.6; 95% confidence interval [CI]: 1.3–2.1), to be prescribed antidepressants or psychotherapy after their CAD diagnosis (aHR: 1.8; 95% CI: 1.2–2.9), or to be hospitalized for an anxiety and depression-related event along with medication or psychotherapy (aHR: 2.0; 95% CI: 1.4–2.9) relative to matched comparisons (n = 2789), during the follow-up period. Patients with primary CAD were at increased risk for medically attended anxiety and depression (aHR: 1.8; 95% CI: 1.4–2.4), with the highest risk for prescription medication or therapy (aHR: 2.7; 95% CI: 1.6–4.6).

**Conclusions:**

Our study indicates that medically attended anxiety and depression manifest at a higher rate in CAD patients than in a matched non-CAD cohort. Study findings suggest that CAD patients may experience a greater burden on mental health that may negatively contribute to their overall quality of life. Further investigation on this topic is warranted.

## Introduction

Cold agglutinin disease (CAD) is a rare and chronic form of autoimmune hemolytic anemia characterized by classical complement pathway–activated hemolysis. CAD accounts for approximately 20% of all autoimmune hemolytic anemia with a prevalence of up to 20 cases per 1 million people [1, 2]. In the United States, the incidence of CAD is estimated at about 1 per 300,000 people and is similar in men and women, with a higher incidence in people who are middle aged and older [3]. The autoantibodies may be idiopathic (primary) or related to an underlying condition such as infection, malignancy, or immune disease (secondary). Clinical manifestations of CAD can include complement-mediated chronic hemolytic anemia and profound fatigue, as well as transient agglutination-mediated circulatory symptoms [4, 5]. CAD in its primary form is a clonal low-grade B-cell lymphoproliferative disorder with no underlying overt malignancy or infection. This is distinguished from cold agglutinin syndrome, in which cold agglutinin titers increase secondary to an underlying condition (such as malignancy or infection). Patients experience unpredictable severe anemia events that require emergency interventions (eg, transfusion) and utilize increased healthcare resources [5–12]. Until recently, there were no approved treatments for CAD; however, sutimlimab was recently approved by the FDA and Japanese health authority after demonstrating efficacy and favorable safety in clinical studies of patients with CAD.

Patients with anemia are more likely to develop anxiety and depression that affect their quality of life [13–16], but there are no studies to date that investigate this in patients with CAD. The aim of this study was to determine the risk of anxiety and depression among patients with CAD using clinical and claims data to classify CAD patients with anxiety and depression, and their outcomes. It is hypothesized that patients with CAD are at an increased risk of developing anxiety and depression.

## Materials and methods

### Study design and data source

We describe, herein, a retrospective matched-cohort comparison study evaluating the risk of medically attended anxiety and depression in patients with and without CAD. All patients with CAD were identified in Optum’s de-identified Integrated Claims-Clinical data set from January 1, 2006 –June 30, 2016. Because of the nature of the data (de-identified), the Optum database does not require IRB approval. This data set links electronic medical record data with adjudicated claims data to provide de-identified information on medications, lab results, vital signs, body measurements, diagnoses, procedures, and clinical notes distilled with Natural Language Processing, for approximately 55 million patients seen throughout the United States. The analytic dataset was complete with no additional linkages conducted by the researchers; there were no missing data.

### Study population and outcomes

Inclusion criteria included CAD patients identified by the International Classification of Diseases (ICD) or medication or therapy, 25 years of age or older, with no history of anxiety and depression. Patients less than 25 years of age at the index date were excluded from the analysis due to the high likelihood that CAD was secondary. CAD patients were identified by the clinical notes with electronically searched terms associated with CAD (“CAD terms”) including “cold agglutinin disease,” “cold autoimmune hemolytic anemia,” or “cold agglutinin hemoglobinuria”. All patients with CAD terms in their clinical notes on one or two separated dates were reviewed by 2 hematologists and were included only after agreement by both physicians. Patients with CAD terms in their clinical notes on at least 3 separate dates were included without further clinical review. The validity of the identification method for patients with CAD terms in their clinical notes on at least 3 separate dates was tested by taking a random sample of 100 patient records with a mention of CAD on at least 3 separate dates. A manual review of these records was conducted by 2 independent hematologists to determine whether the patient had CAD. Agreement was 95% between the hematologists and the initial determination of CAD with the 3-separate date method; thus, the 3-mention criterion was deemed accurate.

Identified CAD patients were matched with up to 10 comparison patients from a 5% random sample of the general population available in Optum. Comparison matches were patients who did not have a CAD diagnosis but had the same sex, age (±3 years), race, region of residence, active time in the Optum health plan, and season and year of entry date into the Optum health plan. Subset analyses were performed further for patients with primary CAD (not due to secondary causes) and their matched comparisons. The outcomes of anxiety and depression were identified based on one of the following events: (1) ICD codes for anxiety and depression only from inpatient stays and outpatient hospital visits, without medication or therapy after index date; (2) medication or therapy without ICD after index date; (3) ICD and at least one medication or therapy after index date; (4) overall medically-attended anxiety and depression: ICD or medication or therapy after the index date, defined as the date the patient was first identified as having CAD in the Optum database. ICD codes for inpatient stays and outpatient hospital visits, medication class, and procedure codes for psychotherapy are detailed in Table 1. If the patient had at least 3 antidepressent (AD) medication codes mentioned on different days, he/she was identified as having anxiety and depression based on medication drug class. If the first medication date was before the index date, the patient was considered to have a history of anxiety and depression. If the first medication date was after the index date, the patient was considered to have new-onset anxiety and depression. The no-event group was defined as no ICD, medication or therapy after the index date. Unmatched subjects were excluded.

**Table 1:** International Classification of Diseases (ICD) medications and procedural codes.

Definition	ICD-10	ICD-9	CPT
**Depression and anxiety codes**			
Depressive individual episodes	F32.x	296.2, 298.0, 625.4, 296.82, 311	
Periodic depression	F33.x	296.3, 296.99, 298.0	
Other anxiety disorders	F41.x	300.0	
Dysthymia, organic depressive disorder	F34.1, F06.32	300.4, 301.12, 293.83	
Persistent affective states: dysthymia, chronic mood disorder of other type, chronic state of mood UNS	F34.1–F34.9	300.4, 301.12, 296.99	
Unspecified affective mental disorder or condition: affective mental disorder UNS	F39	296.9	
Phobic anxiety states: agoraphobia, social anxiety disorder, simple phobia	F40.0–40.2	300.21, 300.22, 300.23, 300.29	
Bipolar disorder in depressive or mixed-mode episodes, other, or UNS	F31.3–F31.9	296.5, 296.6, 296.7, 296.40, 296.45, 296.46, 296.80, 296.89	
**Medication class**			
Antidepressants Selective serotonin reuptake inhibitors Serotonin and norepinephrine reuptake inhibitors Tricyclic antidepressants Selective serotonin reuptake Antidepressants; miscellaneous	N06A		
Antipsychotics	N05A		
Anxiolytics Benzodiazepine Anxiolytics; miscellaneous	N05B		
**Procedure codes**			
Psychotherapy	GZ1, GZ3, GZ5, GZ6, GZ7, GZH, GZJ	94.0, 94.3, 94.4	90832, 90834, 90837, 90791, 90839, 90840, 90845, 90847, 90853

CPT, Current Procedural Terminology; ICD, International Classification of Diseases; UNS, unspecified.

Comorbidities used to build the Charlson Comorbidity Index [17] were accumulated over approximately the same period for the cases and the matched comparison patients, from date of entry into the Optum data set to the index date (see S1 Table for a list of the Charlson Cormorbidies and their ICD codes).

### Follow-up period

The index was considered to be the start of the follow-up period for the CAD patients. The index date of comparison used the same date as matched CAD patients. The patients, at a minimum, must have had at least 1 year of follow-up before the index date and at least 1 month after the index date in the Optum database. Time-to-event was defined as the time from index date till patients had the outcome of anxiety and depression, they left the Optum system, death, or the study period ended (June 30, 2016). Follow-up period is defined as the time from the index date till their last follow-up date in the Optum de-identified Electronic Health Record dataset, death, or June 30, 2016, whichever came first.

### Statistical analyses

Descriptive analyses, including counts, percentages, means, standard deviation (SD), medians, and ranges of patients with CAD and their matched comparisons, were performed for demographic characteristics. Cox regression models, adjusting for age, sex, race, region, Charlson Comorbidity Index score group, and cluster (matched CAD cases and non-CAD comparisons) were used to estimate the hazard ratios for anxiety and depression among patients with CAD versus matched comparators for each of the 4 outcomes. A sensitivity analysis was performed to evaluate the risk of depression among patients presumed to have primary CAD (not due to secondary causes) by excluding patients with CAD with a coexisting diagnosis of any type of lymphoma (except Hodgkin’s disease), myelomas, chronic lymphocytic leukemia, Waldenstrom macroglobulinemia, and certain infections known to be associated with CAD (mycoplasma pneumoniae, Epstein–Barr virus, cytomegalovirus). The comparison patients who were matched to any excluded patients with CAD were removed. The ICD codes used to identify these diagnoses are presented in S1 Table. A further sensitivity analysis excluding benzodiapines was performed to investigate if there was an impact of this class of medications, occasionally prescribed for insomnia, on the overall results. All data management and statistical analyses were carried out using SAS version 9.4 (SAS Institute Inc., Cary, NC, USA).

Some of this work was presented at the 62^nd^ American Society of Hematology annual meeting, 2020 [18].

## Results

There were 814 CAD patients identified in the database between 2006 and 2016 and reviewed for inclusion; a total of 384 patients with CAD (mean [SD] age: 70.0 [13.0] years) who met the anxiety and depression inclusion criteria were included in the analysis (Table 2). Primary reasons for exclusion included anxiety and depression prior to index date (historical anxiety and depression), absence of minimum-required follow-up period before and after index date, and age younger than 25 years. These 384 CAD patients were matched to 2789 comparison patients without CAD (mean [SD] age: 69.6 [12.7] years), with a mean number of matched comparisons per patient of 7.3. The demographic and clinical characteristics of CAD patients and comparisons are shown in Table 2. Over 70% of cases and comparisons were patients 65 years or older (CAD: 75.3%; comparison: 73.1%), and the majority were female (CAD: 57.6%; comparison: 54.4%). The majority of cases (85.2%) and comparisons (84.7%) were Caucasian, and nearly half of both cohorts were from the Midwest.

**Table 2:** Demographic and clinical characteristics of cold agglutinin disease (CAD) patients and their matched comparisons, 2006–2016.

	CAD Patients (N = 384)		Comparisons (N = 2789)	
	N	%	N	%
**Age, years**				
25–64	95	24.7	749	26.9
≥65	289	75.3	2040	73.1
Mean (SD)	70.0 (13.0)		69.6 (12.7)	
Median (range)	72.5 (26–89)		72.0 (25–89)	
**Sex**				
Male	163	42.5	1271	45.6
Female	221	57.6	1518	54.4
**Race**				
White	327	85.2	2362	84.7
Black	14	3.7	175	6.3
Asian	10	2.6	48	1.7
Other/Unknown	33	8.6	204	7.3
**Region**				
Northeast	55	14.3	381	13.7
South	103	26.8	758	27.2
Midwest	172	44.8	1273	45.6
West	48	12.5	344	12.3
Other/Unknown	6	1.7	33	1.2
**Comorbidity (CCI score)**				
Unknown	32	8.3	645	23.1
0	131	34.1	1061	38.0
1–2	158	41.2	865	31.0
3+	63	16.4	218	7.8
**Time to event (months) (ICD hospitalization, Medication, or Therapy)**				
Mean (SD)	27.7 (24.4)		38.2 (25.6)	
Median (range)	20 (0–110)		35 (0–113)	
**Follow-up period. (months)**				
Mean (SD)	33.8 (26.1)		43.5 (26.9)	
Median (range)	28 (1–111)		42 (1–113)	

CAD, cold agglutinin disease; CCI, Charlson Comorbidity Index; SD, standard deviation.

The CCI assesses the number and severity of 19 pre-defined co-morbid conditions associated with increased mortality. Each item receives a score based on its weighting. A score of 0 = no comorbidities; higher scores indicate more numerous and / or more severe conditions, therefore a worse prognosis.

**Fig 1:**
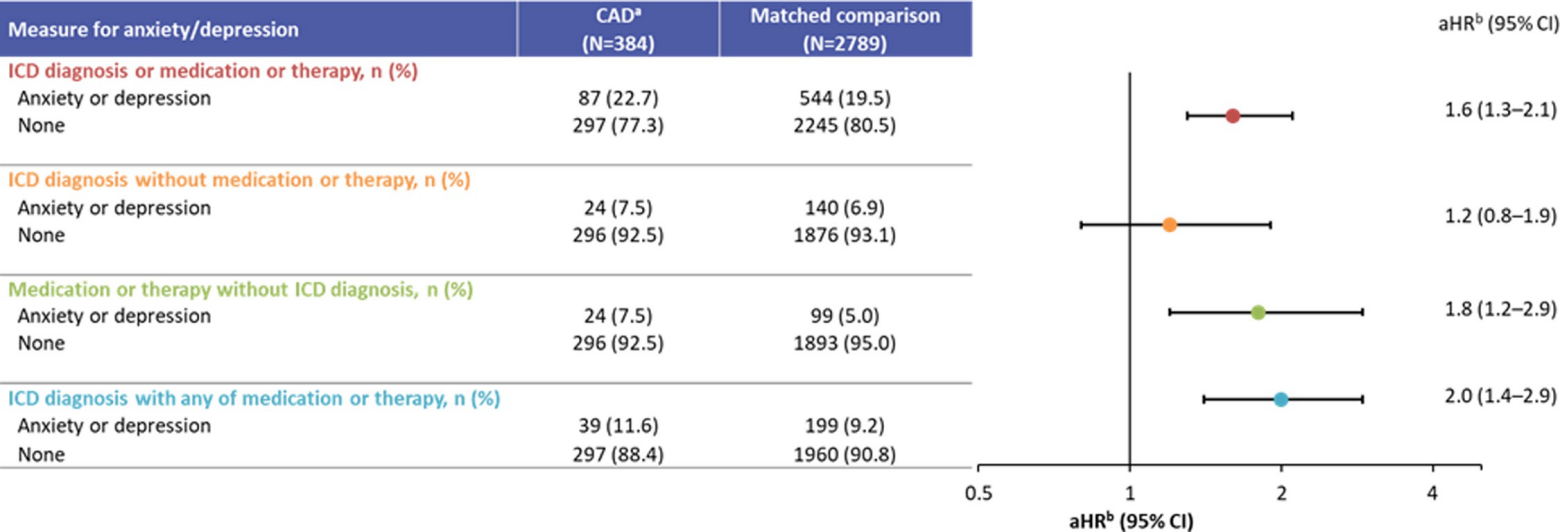
Medically attended anxiety and depression among cold agglutinin disease patients and matched comparisons, 2006–2016. aHR, adjusted hazard ratio; CAD, cold agglutinin disease; CI, confidence interval; ICD, International Classification of Diseases. Median (range) time to first event^c^ was 20 (0–110) months for patients with CAD^a^ and 35 (0–113) months for their matched comparisons. ^a^Included both primary CAD (not due to secondary causes) and secondary cold agglutinin syndrome (coexisting diagnosis of malignancy or infection). ^b^Cox proportional hazard models, adjusted for age, gender, race, region, comorbidity score group, and cluster. ^c^The time to first event was determined as the time from index date (date of diagnosis for both patients with CAD and their matched comparisons) to the confirmation date of the last measure (if >1 measure was used to diagnose anxiety and depression).

CAD patients were at increased risk of medically attended anxiety and depression including outcomes of medication or therapy without inpatient or outpatient visits with an ICD diagnosis for anxiety and depression (adjusted hazard ratio [aHR]: 1.8; 95% confidence interval [CI]: 1.2–2.9), an ICD diagnosis with any of medication or therapy (aHR: 2.0; 95% CI: 1.4–2.9), and ICD diagnosis or medication or therapy (aHR: 1.6; 95% CI: 1.3–2.1), during the follow-up period (Fig 1). There was also a non-significant increased risk for patients with CAD with an inpatient or outpatient visit for anxiety and depression without medication or psychotherapy (aHR: 1.2; 95% CI: 0.8–1.9). The mean (SD) time to event for any hospitalization, medication use, or psychotherapy was 27.7 (24.4) months for patients with CAD and 38.2 (25.6) months for comparisons (Table 2).

**Fig 2:**
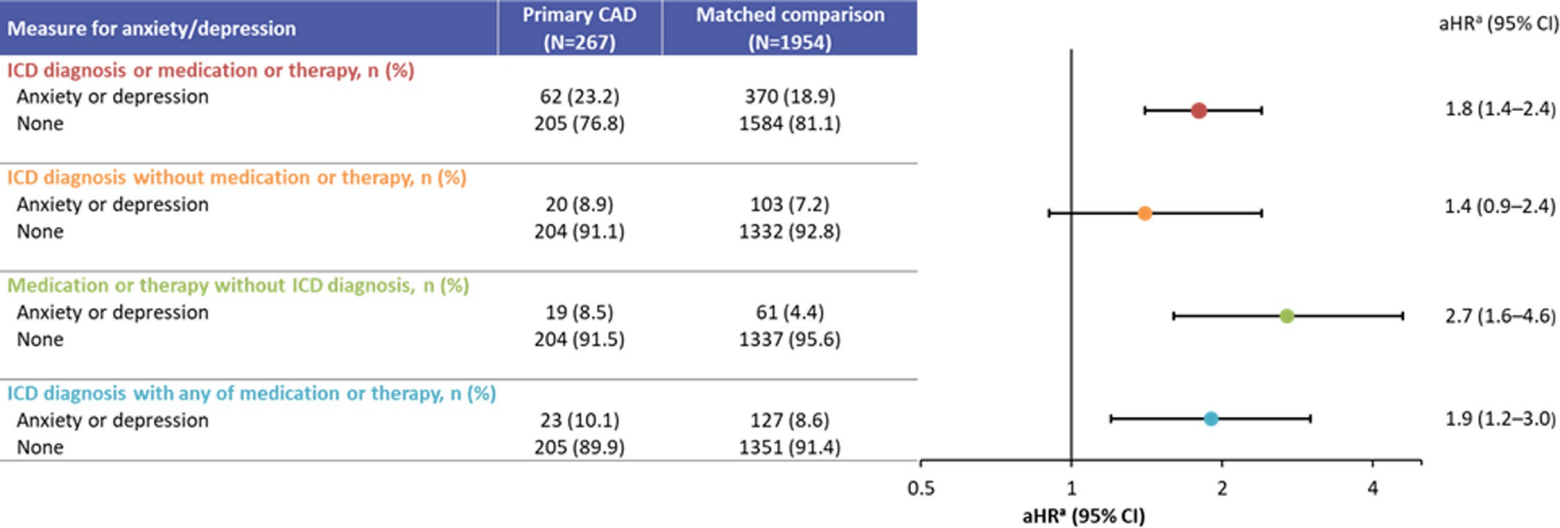
Medically attended anxiety and depression among primary cold agglutinin disease patients and matched comparisons, 2006–2016. aHR, adjusted hazard ratio; CAD, cold agglutinin disease; CI, confidence interval; ICD, International Classification of Diseases. Median (range) time to first event^b^ was 20 (0–110) months for patients with CAD^c^ and 35 (0–111) months for their matched comparisons. ^a^Cox proportional hazard models, adjusted for age, gender, race, region, comorbidity score group, and cluster. ^b^The time to first event was determined as the time from index date (date of diagnosis for both patients with CAD and their matched comparisons) to the confirmation date of the last measure (if >1 measure was used to diagnose anxiety and depression). ^c^Primary CAD only (not due to secondary causes).

In analyses restricted to patients who only had a prescription for medication for anxiety and depression, CAD patients were 1.7 times (95% CI: 1.0–2.8) more likely to use prescription medications for anxiety and depression than comparisons. On average, CAD patients initiated medication for anxiety and depression 15 months (SD: 17 months) after diagnosis. The top 3 medication classes used for CAD patients include benzodiazepines (76%), antipsychotics (24%), and selective serotonin reuptake inhibitors (10%). A similar pattern was seen for comparisons, but the proportion using benzodiazepines (76% vs 60%) was less than for CAD patients. In contrast, use of selective serotonin reuptake inhibitors was higher in the matched comparison group than in CAD patients (31% vs 10%). A similar proportion of CAD patients and comparisons used antipsychotics (24% vs 21%). Excluding benzodiapines from the analysis resulted in similar results (S3 Table). A subanalysis of patients with primary CAD also showed a statistically significant increased risk for medically attended anxiety and depression (aHR: 1.8; 95% CI: 1.4–2.4), with the largest risk for prescription medication or psychotherapy (aHR: 2.7; 95% CI: 1.6–4.6) (Fig 2). The mean (SD) time to event for any hospitalization, medication use, or psychotherapy was 27.0 (24.0) months for patients with primary CAD and 37.6 (24.6) months for comparisons. Patients with primary CAD were almost twice as likely to be hospitalized for anxiety and depression-related events along with medication or psychotherapy (aHR: 1.9; 95% CI: 1.2–3.0). There was also a non-significant risk for patients with primary CAD being hospitalized for anxiety and depression without medication or psychotherapy (aHR: 1.4; 95% CI: 0.9–2.4). Further, analyses restricted to patients who only used medication to treat anxiety and depression found a higher risk for antidepressant use among CAD patients compared with those without CAD (HR: 2.4; 95% CI: 1.3–4.2).

## Discussion

This matched-cohort comparison study aimed to evaluate the risk of medically attended anxiety and depression in patients with and without CAD. Based on our findings, patients with CAD were at increased risk of medically attended anxiety and depression and had a greater healthcare utilization, including increased use of prescribed medication or psychotherapy, with a shorter time to event for any hospitalization, medication use, or psychotherapy. Patients with primary CAD also demonstrated similar results with the highest risk for prescription medication or psychotherapy without ICD diagnosis (aHR: 2.7; 95% CI: 1.6–4.6). To our knowledge, this is the first study to examine the association between CAD and anxiety and depression.

Prior research has demonstrated an association between chronic physical conditions and anxiety and depression disorders [19]. However, few studies have characterized anxiety and/or depression in rare diseases, defined as a group of diseases with a low prevalence (<1:2000) and characterized by heterogeneity. This is related to the challenge of obtaining a large enough sample size in any one rare disease to draw conclusions. One cross-sectional study investigating the summated frequency of anxiety and depression in patients with rare diseases across different diagnoses reported high percentages of clinically relevant symptom burden, including moderately or severely elevated levels in both anxiety (23%) and depression (42%) [20]. In comparison, we found that 23% of CAD patients in our study experienced anxiety and depression (measured by hospitalization, medication, or psychotherapy) after diagnosis.

Mental and physical health are closely linked and have been described in the medical literature as bi-directional. On top of a chronic illness, anxiety and depression can affect the general course of the illness, and increase morbidity and mortality. This can result in an increase in the overall cost of the illness through greater healthcare resource utilization. All of these issues can negatively impact quality of life [21, 22]. This impact may be particularly pronounced in individuals with rare diseases who face additional burdens due to the rarity of their condition, lack of knowledge about the disease, lack of access to specialized medical care, and lack of treatment modalities to manage symptoms of their condition. In another study specifically looking at hematologic rare diseases, myelodysplastic syndromes, aplastic anemia, and paroxysmal nocturnal hemoglobinuria, the scores for depression fell into the mild category, and for anxiety they were moderate [23]. Additional studies on other hematologic diseases that may be classical pathway mediated, such as immune thrombocytopenia (ITP), reveal a high level of feeling anxious; 66% overall felt anxious, with 17% of ITP patients reporting feeling anxious almost all the time. In this study treatment did not seem to impact feelings of anxiety in ITP patients [24].

The potential mechanisms responsible for the concurrent anxiety and depression with chronic diseases are complex and multifaceted, and may involve an interplay of genetic, biological, psychosocial, and behavioral factors. The exact role that complement activation plays in anxiety and depression is unknown, but there are publications suggesting that immune dysregulation leading to a systemic proinflammatory state results in central nervous system imbalance that may potentially contribute to depression [25]. One hypothesis is that a main feature of depression is hypoxia. Hypoxia occurs in patients with autoimmune hemolytic anemia [26], which has a similar profile of symptoms as depression, including headaches and fatigue. Other studies have demonstrated that the pathogenesis of depression may be related to a pro-infammatory state secondary to complement activation [27, 28].

Although many of these theories are speculative regarding the role in inflammation and complement activation systemically and the consequence this may have on central nervous system inflammation and subsequent neuronal degeneration and synapse changes occurring in anxiety and depression, it certainly warrants further investigation; we note a significant difference in manifestation of anxiety and depression in patients with CAD, a disease where persistent classical complement pathway activation is associated with a proinflammatory state [29, 30].

Strengths of the study are the large sample size of individuals and the wide set of potential confounders of the relationship between depression and CAD that were considered. Some limitations are inherent to the study design itself. No causal associations between anxiety and depression and CAD could be drawn. Second, although we adjusted for many factors that may confound the association between CAD and anxiety and depression, other health-related variables not available in the database, including diet and nutritional factors, or inflammatory biomarkers, could not be evaluated. Third, the Optum database only covers commercially insured patients, limiting generalizability to other coverage populations. Finally, our analyses relied on medical codes and there is a potential for misclassification of anxiety and depression using these codes. However, we characterized anxiety and depression using three different outcomes (medication use, hospitalization, and therapy) and all showed a significant excess of anxiety and depression among CAD patients compared to those without CAD. Therefore, it is unlikely that misclassification played a significant role in our findings.

## Conclusions

This study provides the first insight into the association of CAD with anxiety and depression. Our study indicates that medically attended anxiety and depression manifest at a higher rate in patients with CAD than in a matched non-CAD cohort. These findings suggest that patients with CAD may experience a greater burden of disease that potentially has a broader impact on their overall mental health, physical health, and quality of life.

## Supporting information

S1 Table Diagnosis codes for Charlson Comorbidity Index score. (DOCX)

S2 Table Diagnoses excluded for sensitivity analysis of primary cold agglutinin disease. (DOCX)

S3 Table Medically attended anxiety and depression among cold agglutinin disease patients and matched comparisons, 2006–2016. Benzodiazapene use was not considered in these analyses.
(DOCX)
