# Supplementary material for: Medically-attended anxiety and depression is increased among newly diagnosed patients with cold agglutinin disease: Analysis of an integrated claim-clinical cohort in the United States
Source: PLoS One. 2022 Dec 15;17(12):e0276617. doi: 10.1371/journal.pone.0276617 (PMC9754177; doi:10.1371/journal.pone.0276617)
Supplement: S1 Table — (DOCX) [file pone.0276617.s001.docx]

**Supporting information**

**S1 Table.** Diagnosis codes for Charlson Comorbidity Index score

| **Comorbidities** | **ICD-9-CM** | **ICD-10** |
| --- | --- | --- |
| Myocardial infarction | 410.x, 412.x | I21.x, I22.x, I25.2 |
| Congestive heart failure | 398.91, 402.01, 402.11, 402.91,  404.01, 404.03, 404.11, 404.13, 404.91, 404.93, 425.4-425.9, 428.x | I09.9, I11.0, I13.0, I13.2, I25.5, I42.0,  I42.5-I42.9, I43.x, I50.x, P29.0 |
| Peripheral vascular  disease | 093.0, 437.3, 440.x, 441.x, 443.1-443.9, 47.1, 557.1, 557.9, V43.4 | I70.x, I71.x, I73.1, I73.8, I73.9, I77.1,  I79.0, I79.2, K55.1, K55.8, K55.9,  Z95.8, Z95.9 |
| Cerebrovascular disease | 362.34, 430.x-438.x | G45.x, G46.x, H34.0, I60.x-I69.x |
| Dementia | 290.x, 294.1, 331.2 | F00.x-F03.x, F05.1, G30.x, G31.1 |
| Chronic pulmonary  disease | 416.8, 416.9, 490.x-505.x, 506.4, 508.1, 508.8 | I27.8, I27.9, J40.x-J47.x, J60.x-J67.x,  J68.4, J70.1, J70.3 |
| Rheumatic disease | 446.5, 710.0-710.4, 714.0-714.2, 714.8, 725.x | M05.x, M06.x, M31.5, M32.x-M34.x, M35.1, M35.3, M36.0 |
| Peptic ulcer disease | 531.x-534.x | K25.x-K28.x |
| Mild liver disease | 070.22, 070.23, 070.32, 070.33,  070.44, 070.54, 070.6, 070.9, 570.x, 571.x, 573.3, 573.4, 573.8, 573.9, V42.7 | B18.x, K70.0-K70.3, K70.9, K71.3-K71.5, K71.7, K73.x, K74.x, K76.0, K76.2-K76.4, K76.8, K76.9, Z94.4 |
| Diabetes without chronic  complication | 250.0-250.3, 250.8, 250.9 | E10.0, E10.1, E10.6, E10.8, E10.9, E11.0, E11.1, E11.6, E11.8, E11.9, E12.0, E12.1, E12.6, E12.8, E12.9, E13.0, E13.1, E13.6, E13.8, E13.9, E14.0, E14.1, E14.6, E14.8, E14.9 |
| Diabetes with chronic  complication | 250.4-250.7 | E10.2-E10.5, E10.7, E11.2-E11.5, E11.7, E12.2-E12.5, E12.7, E13.2-E13.5, E13.7, E14.2-E14.5, E14.7 |
| Hemiplegia or paraplegia | 334.1, 342.x, 343.x, 344.0-344.6, 344.9 | G04.1, G11.4, G80.1, G80.2, G81.x, G82.x, G83.0-G83.4, G83.9 |
| Renal disease | 403.01, 403.11, 403.91, 404.02,  404.03, 404.12, 404.13, 404.92, 404.93, 582.x, 583.0-583.7, 585.x, 586.x, 588.0, V42.0, V45.1, V56.x | I12.0, I13.1, N03.2-N03.7, N05.2-N05.7, N18.x, N19.x, N25.0, Z49.0-Z49.2, Z94.0, Z99.2 |
| Any malignancy,  including lymphoma  and leukemia, except  malignant neoplasm of  skin | 140.x-172.x, 174.x-195.8, 200.x-208.x, 238.6 | C00.x-C26.x, C30.x-C34.x, C37.x-C41.x, C43.x, C45.x-C58.x, C60.x-C76.x, C81.x-C85.x, C88.x, C90.x-C97.x |
| Moderate or severe liver  disease | 456.0-456.2, 572.2-572.8 | I85.0, I85.9, I86.4, I98.2, K70.4, K71.1, K72.1, K72.9, K76.5, K76.6, K76.7 |
| Metastatic solid tumor | 196.x-199.x | C77.x-C80.x |
| AIDS/HIV | 042.x-044.x | B20.x-B22.x, B24.x |

Quan H, Sundararajan V, Halfon P, et al. Coding algorithms for defining Comorbidities in ICD-9-CM and ICD-10 administrative data. Med Care. 2005;43:1130-9.

ICD-9-CM, International Classification of Disease, Ninth Revision, Clinical Modification

ICD-10, International Classification of Diseases, Tenth Revision.
