# Supplementary material for: Medically-attended anxiety and depression is increased among newly diagnosed patients with cold agglutinin disease: Analysis of an integrated claim-clinical cohort in the United States
Source: PLoS One. 2022 Dec 15;17(12):e0276617. doi: 10.1371/journal.pone.0276617 (PMC9754177; doi:10.1371/journal.pone.0276617)
Supplement: S2 Table — (DOCX) [file pone.0276617.s002.docx]

**Supporting information**

**S2 Table.** Diagnoses excluded for sensitivity analysis of primary cold agglutinin disease.

| **Disease** | **ICD-9** | **ICD-8** | **ICD-10** |
| --- | --- | --- | --- |
| Mycoplasma* | 041.81 | 041.8 (not specific to mycoplasma) | A49.3 |
| Lymphoma |  |  |  |
| Follicular lymphoma | 202.0x | 202.0 | C82.x |
| Non-follicular lymphoma | 200.0x; 200.1x; 200.2x; 200.3x; 200.4x; 200.5x; 200.7x; 200.8x | 200.0; 200.1; 200.2; 200.8 | C83.x |
| Mature T-/NK-cell lymphoma | 200.6x; 202.1x; 202.2x; 202.7x | 202.1; 202.2 | C84.x |
| Other and unspecified types of non-Hodgkin lymphoma | 202.8x | 202.8 | C85.x |
| Other types of T-/NK-cell lymphoma | 202.8 | 202.8 | C86.x |
| HIV disease resulting in Burkitt lymphoma | 200.2x (Burkitt lymphoma) with 042 or V08 or 079.53 (HIV) | 200.2 | C83.7x (Burkitt lymphoma) with a code for HIV (B20; Z21; B97.35) |
| HIV disease resulting in other types of non-Hodgkin lymphoma | 202.8x with 042 or V08 or 079.53 (HIV) | 202.8 | C85.8x with a code for HIV (B20; Z21; B97.35) |
| MALT lymphoma | 200.30 | (No code available) | C88.4 |
| Chronic lymphoid leukemia | 204.1x | 204.1 | C91.1 |
| Waldenström macroglobulinemia | 273.3 | 273.3 | C88.0 |
| Myeloma (any type of myeloma) | 203.0x | 203.0 | C90.x |
| Cytomegalovirus (CMV)* |  |  |  |
| Congenital CMV* | 771.1 | 771.1 | P35.1 |
| Cytomegaloviral mononucleosis* | 078.5 | 078.5 |  |
| Cytomegaloviral disease* | 078.5 | 078.5 | B25.x |

HIV, human immunodeficiency virus; ICD, International Classification of Diseases; MALT, mucosa-associated lymphoid tissue; NK, natural killer.

*Infections were excluded only if the code coincides with the initial CAD diagnosis.
