# Supplementary material for: Medically-attended anxiety and depression is increased among newly diagnosed patients with cold agglutinin disease: Analysis of an integrated claim-clinical cohort in the United States
Source: PLoS One. 2022 Dec 15;17(12):e0276617. doi: 10.1371/journal.pone.0276617 (PMC9754177; doi:10.1371/journal.pone.0276617)
Supplement: S3 Table — Benzodiazapene use was not considered in these analyses. (DOCX) [file pone.0276617.s003.docx]

**Supporting information**

**S3 Table:** Medically attended anxiety and depression among cold agglutinin disease patients and matched comparisons, 2006–2016. Benzodiazapene use was not considered in these analyses.

| ICD without medication or therapy | CAD | | Comparison | | HR  (95% CI) |
| --- | --- | --- | --- | --- | --- |
|  | N | % | N | % |  |
| Anxiety or Depression | 39 | 10.9 | 232 | 9.5 | 1.4  (0.97, 1.9) |
| No Anxiety or Depression | 320 | 89.1 | 2198 | 90.5 |  |

* adjusted by age, gender, race, region, comorbidity score group and cluster

| Medication or Therapy without ICD | Some of the cells had samples that were too small resulting in unreliable results. |
| --- | --- |

| ICD with any of Medication or therapy | CAD | | Comparison | | HR  (95% CI) |
| --- | --- | --- | --- | --- | --- |
|  | N | % | N | % |  |
| Anxiety or Depression | 36 | 10.14 | 173 | 7.3 | 2.2  (1.5, 3.2) |
| No Anxiety or Depression | 319 | 89.9 | 2182 | 92.7 |  |

* adjusted by age, gender, race, region, comorbidity score group and cluster

| ICD or medication or therapy | CAD | | Comparison | | HR  (95% CI) |
| --- | --- | --- | --- | --- | --- |
|  | N | % | N | % |  |
| Anxiety or Depression | 86 | 21.2 | 581 | 18.9 | 1.5  (1.2, 1.9) |
| No Anxiety or Depression | 320 | 78.8 | 2489 | 81.1 |  |

* adjusted by age, gender, race, region, comorbidity score group and cluster
